# Necrotic ulcerated and bleeding striae distensae following bevacizumab in a palliative setting for gliobastomatosis cerebri

**DOI:** 10.3332/ecancer.2017.756

**Published:** 2017-08-07

**Authors:** Olivia Laugier, Laetitia Padovani, Arnauld Verschuur, Caroline Gaudy-Marqueste, Nicolas André

**Affiliations:** 1 Department of Pediatric Hematology and Oncology, AP-HM, Boulevard Jean Moulin, 13005, Marseille France; 2 Department of Radiotherapy, AP-HM, Aix-Marseille Université, Boulevard Jean Moulin, 13005, Marseille, France; 3 Inserm UMR_S 911, Centre de Recherche en Oncologie biologique et Oncopharmacologie, Aix-Marseille Université, Boulevard Jean Moulin, 13005, Marseille, France; 4 Department of Dermatology, AP-HM, Boulevard Jean Moulin, 13005, Marseille, France; 5 OncoSafetyNetwork, AP-HM, Boulevard Jean Moulin, 13005, Marseille, France

**Keywords:** glioblastoma, bevacizumab, steroids, palliative care, skin lesions

## Abstract

Glioblastoma cerebri is a rare paediatric malignancy with dismal prognosis [Chappé C, Riffaud L, and Tréguier C *et al* (2013) **Primary gliomatosis cerebri involving gray matter in pediatrics: a distinct entity? A multicenter study of 14 cases** *Childs Nerv Syst* **29** 565–571 https://doi.org/10.1007/s00381-012-2016-1 PMID: 23306961] and no established standard of care. Here, we report a case of ulcerated and bleeding striae distensae in a teenage girl following palliative treatment with bevacizumab and steroids.

A 15-year-old girl, without previous medical history was referred to our department for investigation in August 2013. Magnetic resonance imaging revealed an infiltrative mass involving both thalami and the temporal lobe. A surgical biopsy was performed and pathological analysis confirmed a diagnosis of WHO grade-IV glioblastoma, so that together with the radiological extent the diagnosis of gliomatosis cerebri was made. The K28M mutation in the histone H3.3 gene (H3F3A) was found. No mutation in IDH1, IDH2, or Braf were found.

No surgical resection was initiated because of both the large size of the tumour and its location. She was initially treated with metronomic temozolomide (50 mg/m^2^/day) in combination with radiotherapy (54 Gy) with subsequent monthly courses of temozolomide 200 mg/m^2^ for 5 days every 4 weeks [2]. Valproic acid as a histone deacetylase (HDAC) inhibitor was added (30 mg/kg/day) to the combination [3]. Clinical and radiological progression were observed 7 months after the end of radiotherapy. A metronomic therapy [4] with oral weekly vinorelbine, daily celecoxib and daily valproic acid was then started and lasted until February 2015 (13 months), when new tumour progression was observed.

She then underwent re-irradiation, followed by bevacizumab (10 mg/kg every 2 weeks) and steroids (prednisolone 1 mg/kg) as a palliative approach. After three injections of bevacizumab, bilateral striae distensae developed on the breasts, inguinal, and axillary areas. Despite bevacizumab discontinuation, the skin lesions rapidly became necrotic and ulcerated and very painful (Figure 1). Management of the skin lesions required prolonged hospitalisation for wound dressing and pain management with morphine administration. The patient died of progressive disease with unhealed striae distensae.

## Discussion

The role of bevacizumab in the treatment of glioblastoma in adults remains controversial [5], although it is frequently used at relapse to try to increase survival. In children with high-grade glioma, bevacizumab does not seem to display efficacy [6] and its role in first-line treatment have been evaluated in the randomised Herby trial [7].

In children with brain tumours treated with bevacizumab toxicities are known, and the risk of defects in wound healing well identified [6], but the occurrence of multiple ulcerated striae distensae is a very rare event [8]. In adults, similar findings have also been reported [9–14] and such cutaneous toxicities very likely result from the combination of steroids and bevacizumab. Patients with a primary brain tumour not uncommonly require chronic corticosteroid therapy and consequently can suffer from striae distensae. The addition of bevacizumab which is known to hamper wound healing [10] likely contributed to the prolonged delayed in wound healing in the patient we report here.

## Conclusion

This case highlights the importance of weighing any benefits with the potential risks associated with palliative anticancer treatment, especially when using new agents outside of clinical trials. Indeed, even if their toxicity profile seems favourable, physicians should be aware of limited knowledge about their toxicity, especially in children. In the case, we report here, in retrospect the side effects associated with the use of bevacizumab ultimately may have outweighed the potential benefits, with significant pain and a prolonged hospital stay. This may justify limiting its use as a palliative approach in children with high-grade glioma.

## Figures and Tables

**Figure 1.**
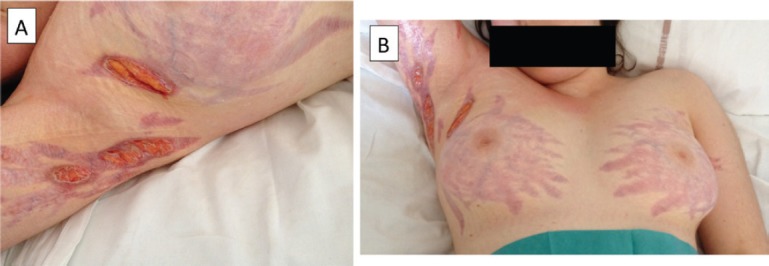
Ulceration of diffuse corticosteroid-induced striae in a teenage patient on bevacizumab for the treatment of advanced glioblastomatosis cerebri.
